# Comparative Study on a Solving Model and Algorithm for a Flush Air Data Sensing System

**DOI:** 10.3390/s140509210

**Published:** 2014-05-23

**Authors:** Yanbin Liu, Dibo Xiao, Yuping Lu

**Affiliations:** 1 College of Astronautics, Nanjing University of Aeronautics and Astronautics, Nanjing 210016, China; E-Mail: nuaa_309@163.com; 2 College of Automation Engineering, Nanjing University of Aeronautics and Astronautics, Nanjing 210016, China; E-Mail: yplac@nuaa.edu.cn

**Keywords:** flush air data sensing system, solving model, measuring precision, real-time feature, neural network

## Abstract

With the development of high-performance aircraft, precise air data are necessary to complete challenging tasks such as flight maneuvering with large angles of attack and high speed. As a result, the flush air data sensing system (FADS) was developed to satisfy the stricter control demands. In this paper, comparative stuides on the solving model and algorithm for FADS are conducted. First, the basic principles of FADS are given to elucidate the nonlinear relations between the inputs and the outputs. Then, several different solving models and algorithms of FADS are provided to compute the air data, including the angle of attck, sideslip angle, dynamic pressure and static pressure. Afterwards, the evaluation criteria of the resulting models and algorithms are discussed to satisfy the real design demands. Futhermore, a simulation using these algorithms is performed to identify the properites of the distinct models and algorithms such as the measuring precision and real-time features. The advantages of these models and algorithms corresponding to the different flight conditions are also analyzed, furthermore, some suggestions on their engineering applications are proposed to help future research.

## 1. Introduction

The design concept of the flush air data sensing system (FADS) was presented by the American National Aeronautics and Space Administration in the 1960s in order to meet the control requirements of the space shuttle [1,2]. FADS has some significant advantages over traditional sensors [3,4], including the higher measurement accuracy and lighter equipment weight. Also, the overall system is installed inside the body such that the vehicle configuration can adapt to the complicated flight environment with consideration of the large angle of attack and high flight dynamic pressure. As a result, FADS can be suitably used in unconventional vehicles such as hypersonic vehicles [5] and Mars entry vehicles [6]. Besides that, compared with the probe type structure, such a built-in stucture makes the vehicle have the lower radar reflective area, leading to the better stealth performance which is critical for the future airplane to escape potential hazards and enhance its survival adaptation [7]. More importantly, further studies on FADS need to be integrated with other technologies such as the vehicle modeling [8], flight control law design [9] and performance evaluation [10] in order to take the advantage of FADS, while improving the overall performance of the unconventional vehicle. Nevertheless, the integrated design of FADS faces some challeges because the relations between the system inputs and outputs exhibit strong nonlinear feartures, and simultaneously the layout of the pressure taps has a significant impact on the measured results. In addition, the useful measurement information is easily affected by external disturbances which are difficult to estimate due to the uncertain and variable flight conditions. Therefore, designing a suitable solving model and algorithm is crucial for FADS to complete the anticipated goals [11].

The current studies on FADS focus on the optimized layout of the pressure taps, the solving model of the air data, the fault detection and reconstruction of the system, the compensation and calibration of air data and so on. In particular, a neural network method was applied in [12] for FADS to achieve measurement of the surface pressure. Similarily, a matrix structure of pressure sensors using neural networks and look-up tables was presented in [13] to estimate the air data of an unmanned air vehicle. In addition, a fault-tolerant neural network algorithm was proposed for FADS in [14], and the self-adaptive and reconstructed capacities can be ameliorated using this algorithm. Furthermore, the neural-network-based model of FADS was developed and demonstrated on a mini air vehicle in [15]. Besides, an improved three-point method was designed in [16] to enhance the solving precision of FADS. Also, the learning of air-data parameters was investigated for FADS in [17]. Furthermore a flush air-data system for transatmospheric vehicles was simulated in [18] to test the overall performances in a real application. Beyond this, the engineering model with regard to the temperature-induced pneumatic sensor was put forward in [19] to satisfy the complicated flight demands under rarefied flow conditions. Moreover, subsonic tests of FADS were conducted for a fixed-wing micro air vehicle to verify the feasibility in the actual flight process [20]. In general, because of the special configuration and complicated intrinsic characteristics for FADS, some new approaches need to be introduced to improve the measurement accuracy and accelerate the convergence rate.

This paper studies comparatively the solving models and algorithms for FADS. Accordingly, there are three aspects which need to be considered. The first question involves the inherent theories of FADS to manifest the relations between the pressure values and air parameters. The second problem relates to the several different solving models and algorithms of FADS which provide useful tools to estimate the necessary air parameters. The third aspect deals with the comparative inverstigation regarding these models and algorithms of FADS in combination of the evaluation criteria, and further by doing the contrastive simulation the results reveal the application characteristics for these distinct models and algorithms.

## 2. Basic Relations between Measuring Pressures and Flight Parameters for FADS

**Figure 1.**
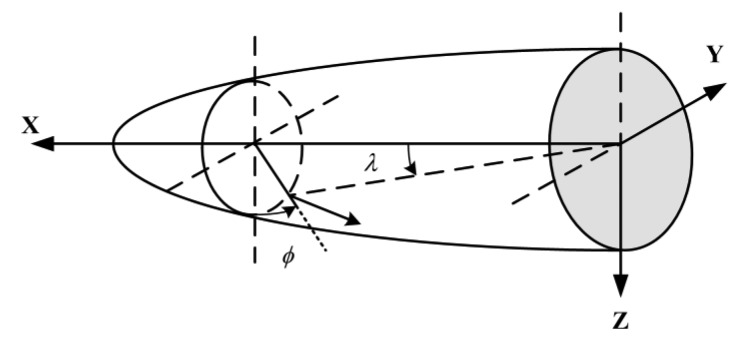
Conical shape applied for FADS.

The work theories of FADS embody that the flight parameters are solved in terms of the pressures measured by the build-in sensors on the vehicle surfaces, whereas these sensors are installed in the given way in relation to the special task [21]. Thus, the distribution of the sensors has a significant impact on the computing accuracy and system redundancy. In this work, we consider the conical shape shown in Figure 1, and this construction is regular such that the pressure taps can be symmetrically placed on the surface.

In Figure 1, *ë* indicates the cone angle, and *ϕ* denotes the circumferential angle. Furthermore, the pressure coefficient using the Newton formula can be written by [13]:

(1) $$\{\begin{array}{c} C_{p}(\theta )=A+B\cos^{2}(\theta )\\ A=\frac{q_{c}}{P_{\infty }}ɛ\\ B=\frac{q_{c}}{P_{\infty }}(1-ɛ) \end{array}$$

where *θ* is the airflow angle of incidence; *ε* indicates the shaped pressure coefficient determined by the flight Mach and airflow angle of incidence; *q_c_, P*_∞_ denote respectively the dynamic pressure and static pressure. Theoretically, the relations of the dynamic pressure q_c_ and the static pressure *P*_∞_ can be decided by the isentropic flow principle, and it is expressed by:

(2) $$C_{1}=\frac{q_{c}}{P_{\infty }}=\left\{ \begin{array}{ll} {\left[1+\frac{\gamma -1}{2}M_{\infty }^{2}\right]}^{\frac{\gamma }{\gamma -1}}-1 & M_{\infty }\leq 1\\ \frac{{\left[\frac{\gamma +1}{2}M_{\infty }^{2}\right]}^{\frac{\gamma }{\gamma -1}}}{{\left[\frac{2\gamma }{\gamma +1}M_{\infty }^{2}-\frac{\gamma -1}{\gamma +1}\right]}^{\frac{1}{\gamma -1}}}-1 & M_{\infty }>1 \end{array}\right.$$

where *γ* is the specific heat coefficient. Based on the momentum and energy conservation principles, we have:

(3) $$C_{p}(0)=\frac{q_{c}}{P_{\infty }}=A+B$$

Furthermore, the pressure coefficient is defined by:

(4) $$C_{p}(\theta )=\frac{p(\theta )-P_{\infty }}{\bar{q}}$$

where *p(θ*) represents the surface pressure. Substituting Equation (4) into Equation (1), we get [14]:

(5) $$p(\theta )=q_{c}(\cos^{2}\theta +ɛ\sin^{2}\theta )+P_{\infty }$$

For any point of the vehicle surface, the airflow angle of incidence *θ_i_* is a function of the angle of attack *α_e_* and the angle of sideslip *β_e_,* and it is expressed by:

(6) $$\cos\theta_{i}=\cos\alpha_{e}\cos\beta_{e}\cos\lambda_{i}+\sin\beta_{e}\sin\phi_{i}\sin\lambda_{i}+\sin\alpha_{e}\cos\beta_{e}\cos\phi_{i}\sin\lambda_{i}$$

Accordingly, the shaped pressure coefficient *ε* is written as:

(7) $$ɛ=f(\alpha_{e},\beta_{e},M_{\infty })$$

where *M*_∞_ denotes the flight Mach, and Equation (7) shows that ε is determined by *α_e_, β_e_* and *M*_∞_, but the special relationship among them is difficult to acquire. On the other hand, this coefficient is critical for FADS to compute the air parameters in terms of the measured pressure values. In fact, Equations (2) and (5)–(7) constitute the solving model of FADS, consisting of four flight state variables in relation to the air datum, namely *α_e_, β_e_, q_c_* and *P*_∞_. In principle, as soon as there are four pressures acquired on the vehicle surface, these variables can be solved accordingly.

## 3. Solving Models and Algorithms of FADS

According to the above relations between the measuring pressures and the flight parameters, we find that it is difficult to obtain the analytical solutions for the corresponding model function due to the nonlinear features. As a result, some solving means are proposed for Equations (2) and (5)–(7), including the least squares method, the three-point method, the neural network method and the look-up table method. These according contents are provided as follows:

### 3.1. Solving Algorithm Based on Least Squares Method

Based on the solving model of FADS in Equations (2) and (5)–(7), the pressure of any point can be expressed by:

(8) $$p_{i}=F_{i}(\theta_{i}(\alpha_{e},\beta_{e}),q_{c},P_{\infty },ɛ(\alpha_{e},\beta_{e},M_{\infty }(q_{c},P_{\infty })))=F_{i}(\alpha_{e},\beta_{e},q_{c},P_{\infty })$$

The linearization model of the above equation can be obtained using the Taylor's expansion mean, and it is expressed by:

(9) $$\begin{array}{l} \begin{array}{c} p_{i}\approx F_{i}(\alpha_{e}^{j},\beta_{e}^{j},q_{c}^{j},P_{\infty }^{j})+{\left(\frac{\partial F_{i}}{\partial \alpha_{e}}\right)}_{\left(\alpha_{e}^{j},\beta_{e}^{j},q_{c}^{j},P_{\infty }^{j}\right)}(\alpha_{e}-\alpha_{e}^{j})+(\frac{\partial F_{i}}{\partial \beta_{e}}){|}_{\left(\alpha_{e}^{j},\beta_{e}^{j},q_{c}^{j},P_{\infty }^{j}\right)}(\beta_{e}-\beta^{j})\\ +(\frac{\partial F_{i}}{\partial q_{c}}){|}_{\left(\alpha_{e}^{j},\beta_{e}^{j},q_{c}^{j},P_{\infty }^{j}\right)}(q_{c}-q_{c}^{j})+(\frac{\partial F_{i}}{\partial P_{\infty }}){|}_{\left(\alpha_{e}^{j},\beta_{e}^{j},q_{c}^{j},P_{\infty }^{j}\right)}(P_{\infty }-P_{\infty }^{j}) \end{array} \end{array}$$

In turn, Equation (9) can be written as:

(10) $$\{\begin{array}{c} \delta {\text{Z}}^{j}={\text{C}}^{j}\;\delta {\text{X}}^{j}\\ \delta {\text{Z}}^{\text{j}}=\left[\begin{array}{l} p_{1}-F_{1}(\alpha_{e}^{j},\beta_{e}^{j},q_{c}^{j},P_{\infty }^{j})\\ \vdots \\ p_{n}-F_{5}(\alpha_{e}^{j},\beta_{e}^{j},q_{c}^{j},P_{\infty }^{j}) \end{array}\right]\\ {\text{C}}^{j}={\left[\begin{array}{cccc} \frac{\partial F_{1}}{\partial \alpha_{e}} & \frac{\partial F_{1}}{\partial \beta_{e}} & \frac{\partial F_{1}}{\partial q_{c}} & \frac{\partial F_{1}}{\partial P_{\infty }}\\ \vdots & \vdots & \vdots & \vdots \\ \frac{\partial F_{n}}{\partial \alpha_{e}} & \frac{\partial F_{n}}{\partial \beta_{e}} & \frac{\partial F_{n}}{\partial q_{c}} & \frac{\partial F_{n}}{\partial P_{\infty }} \end{array}\right]}_{\left(\alpha^{j},\beta^{j},q_{c}^{j},P_{\infty }^{j}\right)}\\ \delta {\text{X}}^{j}={\left[\begin{array}{cccc} \alpha_{e}-\alpha_{e}^{j} & \beta_{e}-\beta_{e}^{j} & q_{c}-q_{c}^{j} & P_{\infty }-P_{\infty }^{j} \end{array}\right]}^{T}\\ \text{X}={\left[\begin{array}{cccc} \alpha_{e} & \beta_{e} & q_{c} & P_{\infty } \end{array}\right]}^{T} \end{array}$$

where *α^j^, β^j^, q_c_^j^, P*_∞_*^j^* denote the *j*th iterative operation points. With further consideration of Equation (5), the according partial derivatives are acquired by:

(11) $$\left\{ \begin{array}{l} \frac{\partial F_{i}}{\partial \alpha_{e}}=2q_{c}\left(\cos\theta_{i}\frac{\partial \left(\cos\theta_{i}\right)}{\partial \alpha_{e}}+ɛ\sin\theta_{i}\frac{\partial \left(\sin\theta_{i}\right)}{\partial \alpha_{e}}\right)+q_{c}\sin^{2}\theta_{i}\frac{\partial ɛ}{\partial \alpha_{e}}\\ \frac{\partial F_{i}}{\partial \beta_{e}}=2q_{c}\left(\cos\theta_{i}\frac{\partial \left(\cos\theta_{i}\right)}{\partial \beta_{e}}+ɛ\sin\theta_{i}\frac{\partial \left(\sin\theta_{i}\right)}{\partial \beta_{e}}\right)+q_{c}\sin^{2}\theta_{i}\frac{\partial ɛ}{\partial \beta_{e}}\\ \frac{\partial F_{i}}{\partial q_{c}}=\cos^{2}\theta_{i}+ɛ\sin^{2}\theta_{i}+q_{c}\sin^{2}\theta_{i}\frac{\partial ɛ}{\partial q_{c}}\\ \frac{\partial F_{i}}{\partial P_{\infty }}=1+q_{c}\sin^{2}\theta_{i}\frac{\partial ɛ}{\partial P_{\infty }} \end{array}\right.$$

For Equation (10), if the number of the pressure taps is more than 4, *δX* can be solved as:

(12) $$\{\begin{array}{c} \delta \text{X}=({\left({\text{C}}^{T}\text{QC}\right)}^{-1}{\text{C}}^{T}){\text{C}}^{T}\text{Q}\delta \text{Z}\\ \text{Q}=\left[\begin{array}{lll} q_{1} & & \\ & \ddots & \\ & q_{i} & \\ & & \ddots \\ & & q_{n} \end{array}\right](n\geq 4) \end{array}$$

where *q_i_* denotes the weighting factor for *i*th pressure tap. In practice, the pressure taps may experience some failures, and in this case *q_i_* corresponding to this bad pressure tap will be set to zero due to the presence of the redundancy. Furthermore, the updated air parameters are described by:

(13) $${\text{X}}^{j+1}={\text{X}}^{j}+\delta {\text{X}}^{j}$$

Repeating the above steps, we can get the resulting solutions with respect to Equation (8). In other words, the necessary air parameters *α_e_, β_e_, q_c_* and *P*_∞_ can be estimated accordingly.

### 3.2. Solving Algorithm Based on Three-Point Method

The air data can be obtained using the three-point method because some special pressure taps can be selected to simplify the FADS model in Equations (2) and (5)–(7). To be specific, three vertical taps along with the leading edge of the central axis are installed to estimate the angle of attack, whereas three pressure taps along with the horizontal axis are used to compute the sideslip angle. Accordingly, the computation equations with regard to these angles are provided by [16]:

(14) $$\{\alpha_{e}=\left\{ \begin{array}{ll} \frac{1}{2}\tan^{-1}\frac{A}{B} & \left|\alpha \right|\leq 45^{{}^{\circ}}\\ \frac{1}{2}(\pi -\tan^{-1}\frac{A}{B}) & \left|\alpha \right|>45^{\circ } \end{array}\right.$$

where:

(15) $$\left\{ \begin{array}{l} A=(P_{k}-P_{j})\sin^{2}\lambda_{i}+(P_{i}-P_{k})\sin^{2}\lambda_{j}+(P_{j}-P_{i})\sin^{2}\lambda_{k})\\ B=(P_{k}-P_{j})\cos\lambda_{i}\sin\lambda_{i}\cos\phi_{i}+(P_{i}-P_{k})\cos\lambda_{j}\sin\lambda_{j}\cos\phi_{j}+(P_{j}-P_{i})\cos\lambda_{k}\sin\lambda_{k}\cos\phi_{k} \end{array}\right.$$

where the subscripts *i, j, k* denote the according label of three pressure taps. Alternatively, the sideslip angle can be acquired. However, these computed angles have multiple values, so the angle of attack can be estimated as the average result of these values, and this is expressed by:

(16) $$\alpha_{e}=\frac{1}{m_{\alpha }}(\alpha_{e1}+\alpha_{e2}+\cdots +\alpha_{em})$$

where *m_α_* represents the number of the gotten angle of attack. Once these angles are determined, the dynamic pressure and the static pressure are obtained based on Equations (5) and (6), and it is given by

(17) $$\left[\begin{array}{c} p_{1}\\ \vdots \\ p_{n} \end{array}\right]=\left[\begin{array}{ll} \cos^{2}\theta_{1}+ɛ\sin^{2}\theta_{1} & 1\\ \vdots & \vdots \\ \cos^{2}\theta_{n}+ɛ\sin^{2}\theta_{n} & 1 \end{array}\right]$$

We see that Equation (17) is difficult to solve due to the existence of *ε*, and thus the iterative computation using the least squares idea is required to deduce *q_c_* and *P*_∞_. Especially, based on Equation (5), the pressure of each tap is reshaped as [16]:

(18) $$\{\begin{array}{c} \text{P}={\text{A}}_{a}{\text{X}}_{a}\\ {\text{A}}_{a}=\left[\begin{array}{ll} 1 & \sin^{2}\theta_{1}\\ \vdots & \vdots \\ 1 & \sin^{2}\theta_{n} \end{array}\right],\text{P}=\left[\begin{array}{c} P_{1}\\ \vdots \\ P_{n} \end{array}\right],{\text{X}}_{a}=\left[\begin{array}{l} {\text{X}}_{a}(1)\\ {\text{X}}_{a}(2) \end{array}\right]=\left[\begin{array}{l} q_{c}+P_{\infty }\\ q_{c}(ɛ-1) \end{array}\right] \end{array}$$

The solution of the least squares with regard to Equation (18) is obtained by:

(19) $${\text{X}}_{a}={{\text{A}}_{a}}^{+}\text{P}$$

Accordingly, the shaped pressure coefficient *ε* is indicated by:

(20) $$ɛ=1+\frac{C_{1}+1}{C_{1}}\frac{{\text{X}}_{a}(2)}{{\text{X}}_{a}(1)}$$

where *C_1_* is decided by Equation (2), and further Equation (20) shows that the shaped pressure coefficient *ε* is gotten for the given *α_e_, β_e_, M*_∞_. Nevertheless, the acquisition of the according solutions is difficult because the relationship among them is strong nonlinear. As a result, the polynomial fitting method is applied to seek this nonlinear relation, and it is provided by:

(21) $$ɛ=A_{0}\left(M_{\infty }\right)+A_{1}\left(M_{\infty }\right)\alpha_{e}+\cdots +A_{m}\left(M_{\infty }\right)\alpha_{e}^{m}+B_{1}\left(M_{\infty }\right)\beta_{e}+\cdots +B_{n}\left(M_{\infty }\right)\beta_{e}^{n}$$

where *A_0_*(*M*),…, *A_m_*(*M*_∞_), *B_1_*(*M*_∞_),…, *B_n_*(*M*_∞_) are the polynomial coefficients related with the flight Mach. Also, Equation (21) tell us that as long as the shaped pressure coefficients are identified in some flight states, the nonlinear expression can be established in accordance with the fitting mean.

### 3.3. Solving Algorithm Based on Neural Network Method

As mentioned above, we know that the expressions between the measuring pressures *P_i_* and the flight parameters *α_e_, β_e_, q_c_, P*_∞_ are difficult to build, but on the other hand their relations can be identified depending on the experimental datum acquired by the tools of computational fluid dynamics. Moreover, the neural network is very suitable to establish such nonlinear connections, and the corresponding structure diagram of the solving algorithm using the neural networks is proposed as shown in the following figure.

**Figure 2.**
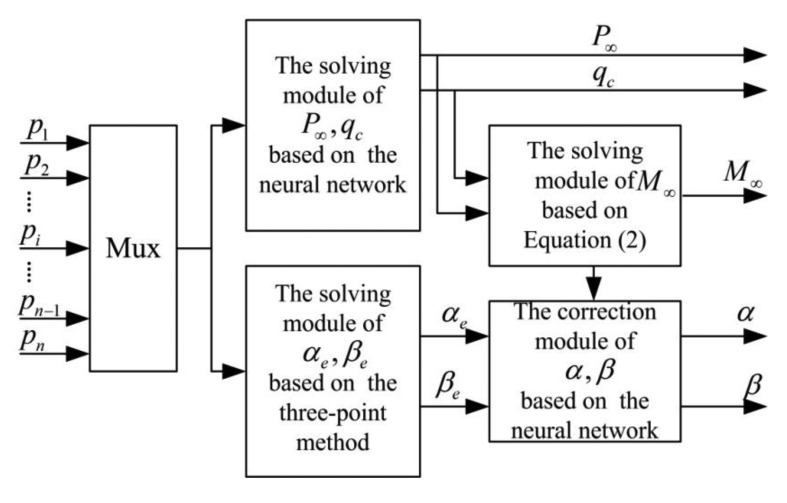
Structure diagram using neural networks for FADS.

In Figure 2, the three-point method is applied to solve *α_e_*, *β_e_*, and then these resulting values are calibrated using the neural network. At the same time, *q_c_*, *P*_∞_ can be computed using the module of the neural network, as a result that this can effectively avoid the complex iteration computation for them. However, these designed neural networks will depend on the large amounts of data, if they are combined with other methods such as the three-point method, the acquired training data may be reduced accordingly, and simultaneously the solving process will be faster due to decreasing the iterative steps [22]. In this paper, the neural network is adopted to compensate the angle of attack and sideslip angle. This is because that the computing course using the three-point method is quick due to without the iteration, but the resulting values in the vicinity of pressure taps tend to have many errors induced by the vehicle itself, for example the unfavorable effects due to the upwash and sidewash actions. Considering these influences, the according corrections for them are necessary to improve the measuring accuracy. Thus, the calibrated relations in Figure 2 are expressed by:

(22) $$\left\{ \begin{array}{l} \alpha +\delta \alpha -\alpha_{e}=0\\ \beta +\delta \beta -\beta_{e}=0 \end{array}\right.$$

where *α_e_*, *β_e_* are respectively the solving angle of attack and sideslip angle by FADS, whereas *α*, *β* represent the angle of attack and sideslip angle corresponding to the free flow. *δα*, *δβ* denote the physical quantities representing the differences between the free stream flow incidence angles and the flow angles as they manifest themselves at the body. Considering the effects of the body induced flow field, *δα* and *δβ* are dependent on the relevant configuration. Thus, the angle errors *δα*, *δβ* can be corrected for FADS using the neural network, we select some training samples acquire by using the CFD tools. In this case, (*α_e_*, *M*_∞_)_1_, …, (*α_e_, M*_∞_)*_κ_* are thought as the inputs of the neural network where κ are the numbers of the training samples, whereas *α_1_*,…,*α_i_* are regarded as the outputs. In the training process, these inputs and outputs will be conducted according to the normalization principle, thus as long as there are enough training samples for FADS, the measuring precision can be guaranteed accordingly.

### 3.4. Solving Algorithm Based on Look-up Method

The core idea of the look-up method is to seek the current air parameters according to predefined databases. Once the pressures of the measuring taps are acquired, the required air parameters can be found directly using these databases. The advantage of this method lies in the rapid solution speed due to the direct look-up course, but on the other hand large amounts of data are required to ensure the system accuracy. For this reason, this method is used with combination of other means, for example, the Mach number is obtained by the inertial navigation system first, and then the angle of attack and the sideslip angle are obtained using the look-up method. Furthermore, the angle of attack can be approximately considered as the proportional relation to the pressure difference, shown as [23]:

(23) $$\alpha_{0}=\left(p_{i}-p_{j}\right)C_{pij}\left(M_{\infty }\right)$$

where *i*, *j* represent respectively the *i*th and *j*th pressure tap. As soon as the sequence of the pressure difference *p_M_* = ([*p_1_* − *p_3_*]_1_,…, [*p_1_* − *p_3_*]*_ê_*) and the sequence of the angle of attack *α_M_* = (*α_M_*,…, *α_ê_*) are known, we have:

(24) $$C_{pij}=p_{M}^{+}\alpha_{M}^{T}$$

Furthermore, with consideration of the *i*th tap or the *j*th tap, Equation (23) is rewritten as:

(25) $$\{\begin{array}{c} \alpha_{1}=p_{i}C_{pi}\left(M_{\infty }\right)\\ \alpha_{2}=p_{j}C_{pj}\left(M_{\infty }\right) \end{array}$$

After averaging these angles of attack, the real angle is estimated by:

(26) $$\alpha =\frac{1}{3}\left(\alpha_{0}+\alpha_{1}+\alpha_{2}\right)$$

In turn, the sideslip angle can also be acquired while identifying the relations between the measuring pressures and the sideslip angles. On the whole, the crucial task of the look-up method is to seek feasible rules corresponding to the inputs and outputs.

## 4. Evaluation Criteria of Solving Model and Algorithm

The above FADS solving models have their respective advantages, thus the selection of the feasible model is important in the practical application. Normally, the evaluation criteria of the solving algorithm include convergence, accuracy, real-time and so on. In particular, the overall layout of the pressure taps has significant impact on the applicability of these solving models, while the number of the pressure taps is connected with the measuring accuracy from the perspective of the system redundant [24]. Based on Equations (7) and (18), we have [16]:

(27) $$\{\begin{array}{c} ɛ=f(\alpha_{e},\beta_{e},M_{\infty })=f(\alpha_{e},\beta_{e},G^{-1}(\frac{q_{c}}{P_{\infty }}))\;=f(\alpha_{e},\beta_{e},G^{-1}(\frac{1}{t_{a}(ɛ-1)-1}))\;\\ t_{a}=\frac{{\text{X}}_{a}(1)}{{\text{X}}_{a}(2)} \end{array}$$

where *G* is determined by Equation (2). Let |*f′_ε_*| = *h*(*α*, *β*, *M*_∞_), then this yields:

(28) $$h(\alpha ,\beta ,M_{\infty })=\left|\frac{\partial f(\alpha ,\beta ,M_{\infty })}{\partial ɛ}\right|=\left|\frac{\partial f}{\partial M_{\infty }}\frac{\partial M_{\infty }}{\partial \left(q_{c}/P_{\infty }\right)}\frac{\partial \left(q_{c}/P_{\infty }\right)}{\partial ɛ}\right|$$

According to Equations (2), (18) and (20), we can get:

(29) $$\frac{\partial M_{\infty }}{\partial \left(q_{c}/P_{\infty }\right)}=\frac{1}{\overset{\partial \left(q_{c}/P_{\infty }\right)}{\;}/\underset{\partial M_{\infty }}{\;}}=\left\{ \begin{array}{ll} \frac{1}{1.4M_{\infty }{\left(1+0.2M_{\infty }^{2}\right)}^{2.5}} & M_{\infty }<1\\ \frac{7{\left(M_{\infty }^{2}-1\right)}^{3.5}}{1168.44M_{\infty }^{6}(2M_{\infty }^{2}-1)} & M_{\infty }>1 \end{array}\right.$$

(30) $$\frac{\partial \left(q_{c}/P_{\infty }\right)}{\partial ɛ}=-\frac{{\text{X}}_{a}\left(2\right){\text{X}}_{a}\left(1\right)}{{\left({\text{X}}_{a}\left(1\right)\left(ɛ-1\right)-{\text{X}}_{a}\left(2\right)\right)}^{2}}=\frac{\left(q_{c}/P_{\infty }\right)\left(q_{c}/P_{\infty }+1\right)}{1-ɛ}$$

Substituting Equations (29) and (30) into Equation (28), we have:

(31) $$h(\alpha ,\beta ,M_{\infty })=\left|\frac{\left(q_{c}/P_{\infty }\right)\left(q_{c}/P_{\infty }+1\right)\frac{\partial f}{\partial M_{\infty }}}{(1-ɛ)\frac{\partial \left(q_{c}/P_{\infty }\right)}{\partial M_{\infty }}}\right|=\left|\frac{g(M_{\infty })[g(M_{\infty })+1]\frac{\partial f}{\partial M_{\infty }}}{[1-f(\alpha_{e},\beta_{e},M_{\infty })]\frac{\partial [q_{c}/P_{\infty }]}{\partial M_{\infty }}}\right|$$

From Equations (28) and (31), we find that as long as *h*(*α*, *β*, *M*_∞_) ≤ 1, Equation (27) is convergent such that the shaped pressure coefficient *ε* can reach the respected value using this iteration method. Beside the convergence, the measuring accuracy is the other criteria required to consider. Thus, based on Equations (2), (5) and (18), the dynamic pressure and static pressure can be approximately estimated by:

(32) $$\{\begin{array}{c} q_{c}=\frac{C_{1}X_{a}(1)}{1+C_{1}}\\ P_{\infty }=\frac{X_{a}(1)}{1+C_{1}} \end{array}$$

Furthermore, the error percentages corresponding to the dynamic pressure and the static pressure are expressed by:

(33) $$\{\begin{array}{c} e_{q}=\frac{\left(q_{c}-q_{ctrue}\right)}{q_{ctrue}}\times 100\%\\ e_{P}=\frac{\left(P_{\infty }-P_{ctrue}\right)}{P_{ctrue}}\times 100\% \end{array}$$

where *q_ctrue_*, *P_ctrue_* represent respectively the resulting values acquired using the CFD tools. From Equation (18), we know that the dynamic pressure and static pressure can be adjusted by altering the shaped pressure coefficient *ε*, but on the other hand their sum is fixed. In other word, once *X_α_(1)* is solved, the total error of the dynamic pressure and static pressure is identified, so no matter how to adjust *ε*, the according error exists. In principle, this error can be eliminated as *q_ctrue_*, *P_ctrue_* are integrated to the solving algorithm, as a result that the further calibration is required for FADS to ameliorate the measuring accuracy [25].

Beyond these, the real-time characteristics with regard to these algorithms need to be taken into account. In common, the iterative process will bring the unfavorable time delay, and thus the operation speed of the solving model based on the neural network or the look-up methods is faster than that based on the three-point method. However, there is the fundamental contradiction between the real-time feature and solving precision, so a compromise is necessary for the performance evaluation of the solving model. In addition, the selection of the initial values will have significant effects on the real-time characteristics because the iteration process will stop rapidly as the initial values are given well.

## 5. Simulation Study and Comparative Analysis

The shaped pressure coefficient *ε* is critical to solve the air parameters, but according to Equation (7), it is related with the angle of attack, sideslip angle and flight Mach. Therefore, *ε* needs to be identified first. Based on the three-point method, as long as the pressures are obtained, the local angle of attack and local sideslip angle can be computed accordingly. After that, *ε* is obtained in accordance with Equations (18)–(20), while considering the current flight Mach. In the simulation, for the body surface in Figure 1, the pressure data of the measuring taps corresponding to *M*_∞_ = 2.2, 2.5, 2.75, 3, can be acquired by using the CFD tool, so the change curves of the shaped pressure coefficient ε are provided as follows.

**Figure 3.**
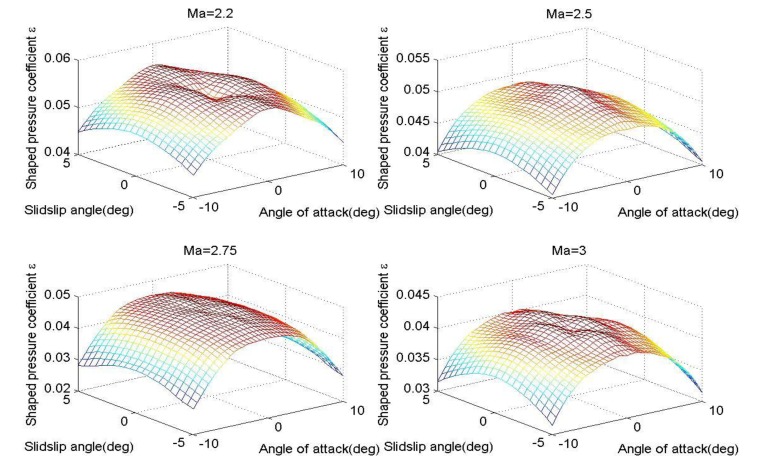
Change curves of shaped pressure coefficient.

Figure 3 shows that the relations between the shaped pressure coefficient and the according flight parameters, and such results reflect the nonlinear features associated with Equation (7). Furthermore, using these different solving algorithms, the angle errors of attack between the solving results and the CFD data are displayed as follows.

**Figure 4.**
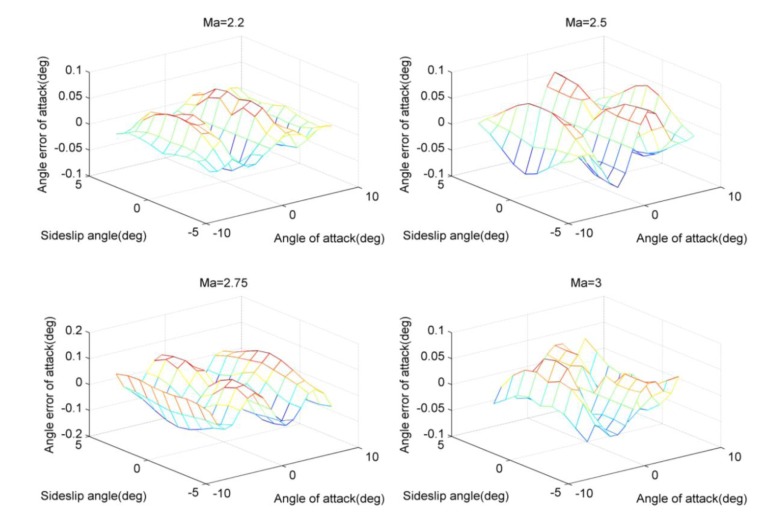
Change curves of angle errors of attack using least squares method.

**Figure 5.**
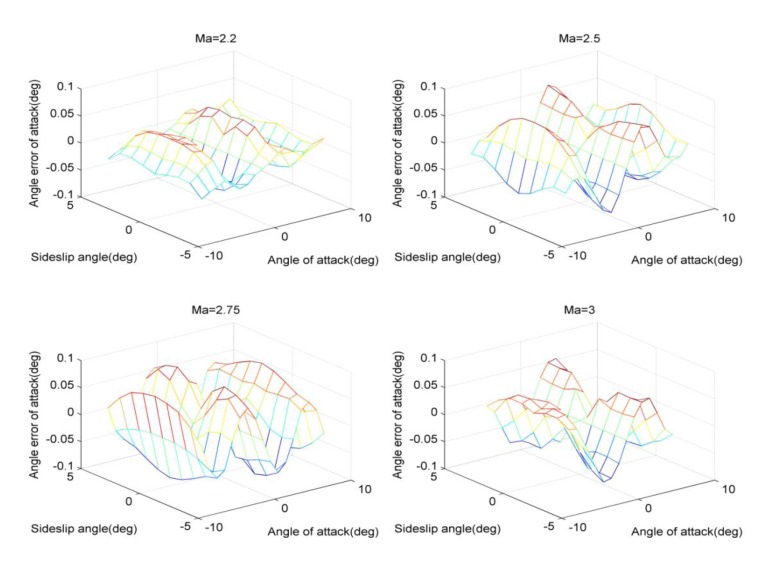
Change curves of angle errors of attack using three-point method.

Figures 4 and 5 demonstrate the absolute value of the angle errors of attack are less than 0.1 degree using the calibrating compensation. This reflects that the application of the least squares method or the three-point method will cause the considerable errors due to the inaccurate modeling process. Thus, the angle correction may be effective to improve the measuring accuracy. Furthermore, the neural network and the look-up method are applied, and the according results are provided in the following figures.

**Figure 6.**
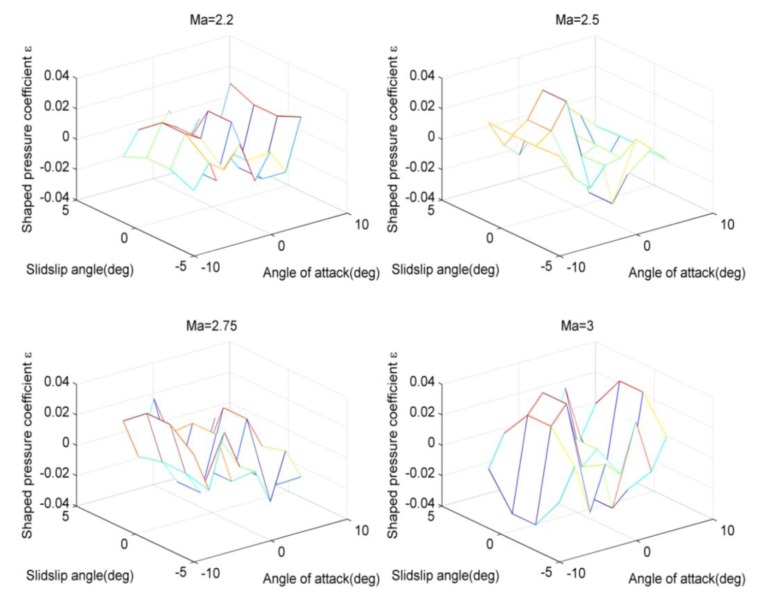
Change curves of angle errors of attack using neural network.

**Figure 7.**
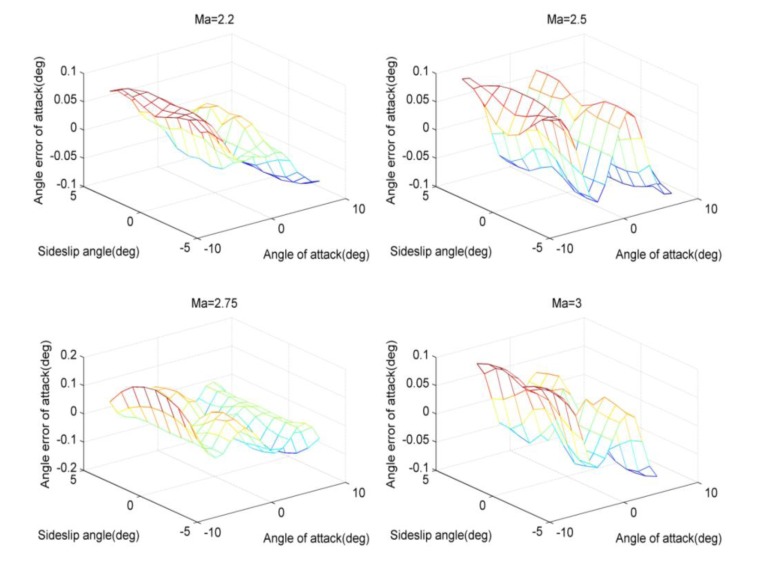
Change curves of angle errors of attack using look-up method.

From Figure 6, we know the solving errors reach less than 0.05 when the neural network method is adopted, and this is because that such a process depends on the CFD data instead of the special model, so accordingly the modeling errors are very small. Additionally, Figure 7 shows that the angle errors of attack using the look-up method is larger than that using the neural network due to the lack of the training course, but the operation speed is very rapid with consideration of the direct solution procedure. Correspondingly, for the body surface in Figure 1, the comparative results of the different solving methods are listed in Table 1.

Table 1 demonstrates that the solving precision will improve after using the according correction, and the three-point method can get better results in contrast to the least squares method. Among them, the solving errors are smallest using the neural network. As a result, if the measuring accuracy is thought as the most important item, the solving model based on the neural network is ideal to realize the anticipated goals.

To be specific, the solving algorithm based on the neural network can ensure the robustness of the resulting calculation as the training model is identified. This is because that such a built model is obtained using the large amount of sampling data, so several inaccurate samples may have less impact on the solving accuracy. In principle, system robustness can be improved if more sampling data can be provided. Nevertheless, training more sampling data requires more time, leading to the real-time reduction. As a result, getting the proper amount of exact samples will be crucial for the neural network algorithm to guarantee system robustness, as well as the computation efficiency. On the other hand, the following solving process is subjected to this trained model, so the inputs lying in the range of the samples will result in the more accurate results, whereas the deviation from the sample range will deteriorate the solving precise. Therefore, the solving algorithm based on the neural network should be adopted in the vicinity of the samples input range. Furthermore, the real-time characteristics are considered in the simulation, and the durations of the solving process using these algorithms are shown in Table 2.

Table 2 tells us that the duration of the neural network is smaller than that of the least squares method or the three-point method, whereas the time-consuming value using the look-up method is minimal among them due to without the iteration and training process. However, the look-up method has the less robust performance because the solving process is based on simple interpolation. Thus, once the pressure inputs significantly differ from the given database, the unfavorable results such as the discontinuous flutter may emerge, as a result that the solving algorithm fails to operate. Normally, the output results of the look-up method are satisfactory if the pressure inputs are matched with the provided samples. On the contrary, as soon as the inputs are far from these sampling points, the solving accuracy will decrease accordingly. Consequently, the trade-off consideration among the different criteria is a prerequisite in real applications, and the efficient and applicable demands are important for FADS to implement the complicated tasks.

## 6. Conclusions

This paper deals with comparative studies of the different solving models for FADS. First, the basic connection between the measuring pressures and flight parameters is given to demonstrate the strong nonlinear features among them. Then, the solving models and algorithms of FADS are provided using the least squares method, three-point method, neural network method and look-up method. Afterwards, the evaluation criteria of these models and algorithms are introduced for FADS. Furthermore, simulation work is conducted to comparatively analyze the feasibility of these FADS solving models. We believe the work in this paper will provide the helpful information for FADS studies to meet the complicated task demands in the future.

## Figures and Tables

**Table 1.** Comparative results of different solving methods.

		**Angle Bias of Attack (°)**	**Sideslip Angle Bias (°)**	**Dynamics Pressure Bias (%)**	**Static Pressure Bias (%)**	**Mach Bias (10^−3^)**
Least Squares Method	Without calibration	1.7004	0.8497	1.7894	1.7894	0.9089
	With calibration	0.1004	0.0838	0.4297	0.4305	0.2434

Three-point Method	Without calibration	1.6244	0.8901	1.7894	1.7893	0.8474
	With calibration	0.0877	0.1049	0.4250	0.4291	0.2356

Neural Network		0.0395	0.0971	0.0113	6.717 × 10^−11^	0.3090

Look-up Method		0.1626	0.1649	0.6432	0.6834	0.2845

**Table 2.** Durations of solving process with regard to different algorithms.

**Least Squares Method (s)**	**Three-Point Method (s)**	**Neural Network (s)**	**Look-Up Method (s)**
271.5641	145.5505	5.1184	0.1132

## Nomenclature

λ: The cone angle
*ϕ*: The angle bias of attack
*θ*: The airflow angle of incidence
*ε*: The shaped pressure coefficient
*q_c_*: The dynamic pressure
*P*_∞_: The static pressure
*γ*: The specific heat coefficient
*α_e_*: The angle of attack
*β_e_*: The angle of sideslip
*δα*: The angle bias of attack
*δβ*: The angle bias of sideslip
*Q*: The weighting factor matrix
*q_ctrue_*: The true value of the dynamic pressure
*P_ctrue_*: The true value of the static pressure
*e_q_*: The error percentage of the dynamic pressure
*e_p_*: The error percentage of the static pressure
*m_α_*: The number of the gotten angle of attack
*M*_∞_: The flight Mach
